# Serum Penicillin G Levels Are Lower Than Expected in Adults within Two Weeks of Administration of 1.2 Million Units

**DOI:** 10.1371/journal.pone.0025308

**Published:** 2011-10-04

**Authors:** Michael P. Broderick, Christian J. Hansen, Kevin L. Russell, Edward L. Kaplan, Jeffrey L. Blumer, Dennis J. Faix

**Affiliations:** 1 Henry M. Jackson Foundation for the Advancement of Military Medicine, Naval Health Research Center, San Diego, California, United States of America; 2 Naval Health Research Center, San Diego, California, United States of America; 3 United States Department of Defense Global Emerging Infections Surveillance and Response System, Silver Spring, Maryland, United States of America; 4 Department of Pediatrics, University of Minnesota Medical School, Minneapolis, Minnesota, United States of America; 5 Departments of Pediatrics and Pharmacology, Case Western Reserve University School of Medicine, Cleveland, Ohio, United States of America; University of South Dakota, United States of America

## Abstract

When introduced in the 1950s, benzathine penicillin G (BPG) was shown to be effective in eradicating group A beta-hemolytic streptococcus (GAS) for at least 3 weeks after administration. Several studies since the 1990s suggest that at 3–4 weeks serum penicillin G levels are less than adequate (below MIC^90^ of 0.016 µg/ml). We studied these levels for 4 weeks after the recommended dose of BPG in military recruits, for whom it is used as prophylaxis against GAS. The 329 subjects (mean age 20 years) each received 1.2 million units BPG IM and gave sera 1 day post injection and twice more at staggered time points over 4 weeks. Serum penicillin G levels were measured by liquid chromatography/tandem mass spectometry. The half-life of serum penicillin G was 4.1 days. By day 11, mean levels were <0.02 µg/ml, and by day 15<0.01 µg/ml. Levels in more than 50% of the subjects were below 0.02 µg/ml on day 9, and <.01 µg/ml on day 16. There was no demonstrable effect of subject body-surface area nor of the four different lots of BPG used. These data indicate that in healthy young adults serum penicillin G levels become less than protective <2½ weeks after injection of 1.2 million units of BPG. The findings require serious consideration in future medical and public health recommendations for treatment and prophylaxis of GAS upper respiratory tract infections.

## Introduction

Benzathine penicillin G (BPG) has been a mainstay of treatment and prophylaxis against group A beta-hemolytic streptococcus (GAS) since its clinical introduction in the early 1950s [1]. Studies by Rusoff and Stollerman [2] demonstrated that this repository form of penicillin provided adequate serum levels for both treatment of GAS as well as for prevention of acquisition of group A streptococci for 3 weeks or longer following injection at recommended BPG doses.

BPG remains an integral part of most recommendations/guidelines for management of GAS upper respiratory tract infections [3], [4], [5]. Its primary advantages include improved compliance over oral preparations, and improved effectiveness even when compliance has been documented [6]. It has proved effective in both endemic and epidemic GAS disease, especially in military populations where streptococcal infections have historically been and remain significant medical and public health problems.

Recently, unexpectedly high streptococcal treatment failure rates were reported when treating streptococcal pharyngitis with BPG [6]. The duration of adequate serum penicillin levels for GAS is dose-dependent; the larger the dose of BPG, the higher the serum concentration for longer periods of time [7]. A BPG study by Bass et al. [8]—the only relatively recent study (1996) in otherwise healthy adults—reported that serum penicillin levels were below assayable levels at less than three weeks following BPG injection in healthy military trainees. We performed a meta-analysis that indicates that there has been, since the studies of the 1990s, a reduction in the probability that at 3 weeks a protective level of BPG serum will be maintained, whether expressed by percentage of subjects above the anticipated serum penicillin levels or by means of serum penicillin levels in study subjects (Broderick, Faix, and Hansen, unpublished data).

Failure of microbiological treatment and faster-than-expected clearance in light of unchanged susceptibility of GAS to penicillin [9], [10] hamper the effective use of currently available BPG. If current formulations of BPG result in pharmacokinetic profiles that result in lower than anticipated levels relative to the past reports, the adverse consequences for BPG treatment and prophylaxis would be significant [7].

Standard guidelines for use of BPG are intramuscular administration of 1.2 million units initially and every 3or 4 weeks, depending on the nature of the illness and the health status of the individual [3]. The purpose of this study was to examine the pharmacokinetics of the currently available intramuscular BPG preparation in the United States in a high-risk population.

## Methods

### Ethics statement

The study was approved by the Naval Health Research Center institutional review board (protocol number NHRC.2007.0022). All participants provided informed written consent.

### Subjects

In January 2008, 164 newly reporting military trainees were enrolled in the study after being briefed on study procedures and providing written informed consent. In March 2008 an additional 165 subjects were enrolled. Each of these two cohorts was followed for 29 days after enrollment. Enrollment was limited to male trainees (no females train at the military training facility) who were not allergic to penicillin and had no history of rheumatic fever or rheumatic heart disease. None had received antibiotics recently.

### Procedure

On day 0, as part of standard military medical processing for initial entry training, subjects were administered an intramuscular (gluteal) dose of 1.2 million units of BPG (Monarch Pharmaceuticals [subsidiary of King Pharmaceuticals] L-A 2 ml Bicillin; NDC number 60793070110; mfg. cat. no. 1138883). Doses from four manufactured lots were equally divided among the subjects and the lot number was recorded for each subject. No additional BPG doses or other penicillin or penicillin related antibiotics were administered during the study. Four percent of the subjects received a non-penicillin-type of antibiotic some time during the study which would not have been detected. Body-surface area was calculated for each subject using self-reported height and weight [11].

The protocol was identical for each cohort. Each subject gave blood three times. The first time for all subjects was approximately 24 hours after the BPG injection. The subjects were then randomly assigned to 1 of 10 groups of between 24–36 subjects which were kept intact for their two subsequent blood draws. The groups were staggered for administration of the blood draws as shown in Table 1.

**Table 1:** Plan for blood draws.

Group	BPG doseall on day 0	Draw 1all on day 1	Draw 2	Draw 3
1			Day 3	Day 16
2			Day 4	Day 17
3			Day 6	Day 20
4			Day 7	Day 21
5	Day 0	Day 1	Day 8	Day 22
6			Day 9	Day 23
7			Day 10	Day 24
8			Day 13	Day 27
9			Day 14	Day 28
10			Day 15	Day 29

Each cohort had 10 groups, and each group had between 12 and 18 subjects.

Each blood draw consisted of two 10 ml serum-separator tubes. The blood was allowed to clot at room temperature for 20 minutes, centrifuged at 2000 rpm for 15 minutes, and serum transferred into two 4 ml cryogenic vials labeled with the subject's study ID and date of collection; all samples were then stored at −70°C.

### Laboratory analysis

The sera were analyzed (blinded to subject, date, and order of collection) for penicillin G levels using liquid chromatography mass spectroscopy (LC/MS/MS). A 1.0 mL aliquot of serum was extracted with 4.0 mL of acetonitrile. After vortexing and centrifugation the organic layer was transferred to a clean tube, dried with a stream of nitrogen at 40°C, then reconstituted in mobile phase for injection a Varian 1200L LC/MS/MS in the ESI (electrospray ionization) mode. Separation on the high pressure liquid chromatography was achieved using a Polaris 5 µ C18-A, 50×2.0 mm column (Sigma-Aldrich, St. Louis, MO). The mobile phase was A: 0.1% Acetic Acid and B: methanol +0.1% acetic acid with a gradient of 10% to 50% B in 8 minutes. The internal standard penicillin V was measured by single reaction monitoring. Penicillin G was measured at the 335.1 (parent mass) transition to 160.0 (fragment mass) and the internal standard at the 365.1(parent mass) transition to 160.0 (fragment mass). The limit of quantitation of this procedure was 500 pg/ml.

### Statistical analysis

Analysis of variance (ANOVA) was performed to evaluate differences in serum levels between the two cohorts, among the four lots of BPG, and among quartiles of body surface size, and to examine the relationship of each of these factors with day since injection. Mean serum penicillin G levels and proportion of subjects above putative protection levels were calculated for each day. Simple linear regression was used to evaluate the relationship between body surface area and serum penicillin G concentrations on the first day after injection. Where no difference was noted between cohorts they were collapsed for analysis. The least-squares method was used to fit an exponential curve to the daily measurements and to estimate the half-life of serum penicillin G following the injection.

We made no assumptions regarding the kinetics of serum penicillin levels and sampled our study population throughout the 29-day period of observation. This provided a continuous profile of serum penicillin G levels, rather than the limited time points that have been reported in most previous studies.

## Results

A total of 329 male subjects (mean age = 20 years [Table 2]) were enrolled; a total of 953 blood samples (average of 2.9 samples per subject) were collected.

**Table 2:** Demographics of study subjects.

Demographics		Cohort 1 mean (range)	Cohort 2 mean (range)	Overall mean (range)
No. of subjects		*n* = 164	*n* = 165	n = 329
Age (y)		20, 17–27	20, 18–32	20, 17–32
Height (in)		70, 63–76	69, 57–77	70, 57–77
Weight (lb)		167, 110–240	170, 115–237	169, 110–240
Body surface area groups by quartile (sq m)	Quartile 1	1.52–1.81	1.53–1.82	1.52–1.81
	Quartile 2	1.82–1.92	1.83–1.93	1.82–1.92
	Quartile 3	1.93–2.05	1.94–2.04	1.93–2.05
	Quartile 4	2.06–2.41	2.05–2.61	2.05–2.61

There were no significant differences in serum levels between cohorts. One subject whose serum never showed detectable penicillin levels was removed from analysis since the study was designed to measure changes in detectable levels. The cause for this could not be determined. One subject had serum penicillin G levels consistent with the other subjects at days 1 (0.073 µg/ml) and 8 (0.003 µg/ml) but had an extremely high value for day 22 (0.1485 µg/ml vs. mean of 0.006 and range 0 to 0.026 for the rest of the subjects). This apparent outlier was excluded from the day 22 mean penicillin G serum level calculation.

ANOVA found no significant difference between the two enrollment cohorts or the four BPG lots (data not shown). There was no evidence of interaction between cohort, BPG lot or body size and day since injection. Day since injection was the only significant contributor to serum penicillin G level.

**Figure 1:**
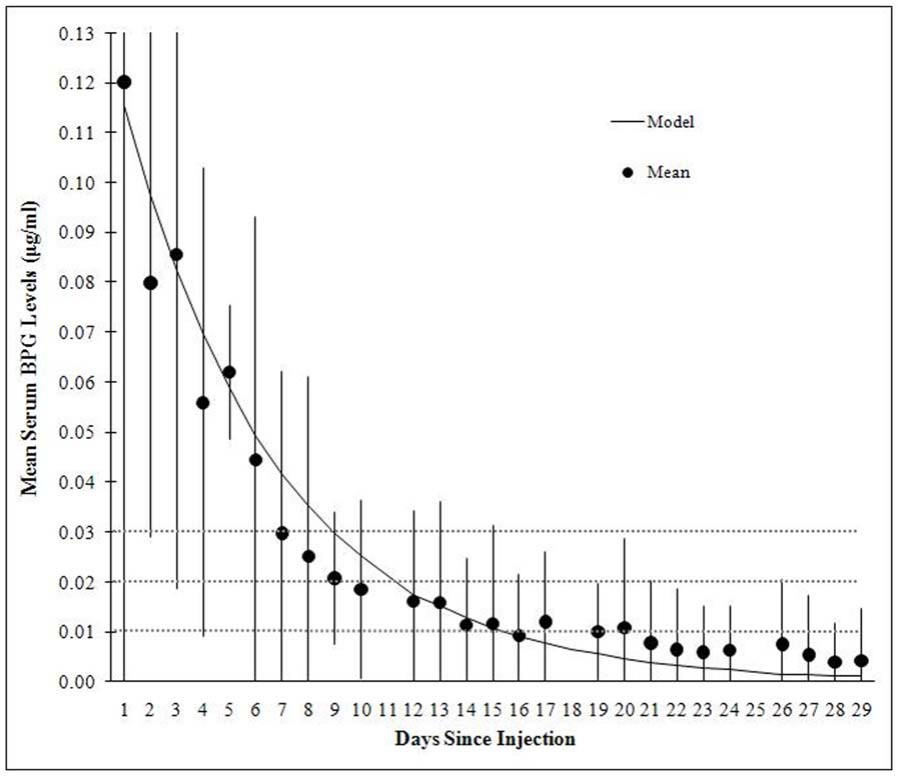
Mean BPG level and 95% confidence interval versus day since injection. Each subject provided a sample 1 day after injection and two more over the course of 28 days. The serum penicillin G half life is 4.1 days. At approximately day 11 the expected mean penicillin G serum level is less than 0.02 µg/ml. On days 1, 2 and 3 the 95% confidence intervals extend upward beyond 0.13 and are truncated to allow more visual resolution at the lower end of the curve. (Note: day 21 outlier removed from calculation of mean; see text.).

The half-life of serum penicillin levels for the study subjects was 4.1 days in the exponential model. Table 3 and Figure 1 show the relationship between mean daily serum penicillin G levels and their 95% confidence intervals. The mean serum penicillin level fell below putative protection levels at different days. From day 9 on, the mean level was less than 0.03 µg/ml; from day 11 on the mean level was less than 0.02 µg/ml; and from day 15 on the mean was less than 0.01 µg/ml.

**Table 3:** Mean serum penicillin G levels and 95% confidence intervals for days since injection.

Days sinceinjection	*N*	Mean(µg/ml)	Confidenceinterval (µg/ml)
1	325	0.119	0.0000.259
2	4	0.080	0.029–0.131
3	25	0.086	0.019–0.152
4	32	0.056	0.013–0.099
5	4	0.062	0.049–0.075
6	31	0.043	0.000–0.092
7	36	0.030	0.001–0.059
8	27	0.025	0.000–0.059
9	32	0.021	0.007–0.034
10	32	0.018	0.002–0.035
12	7	0.016	0.000–0.034
13	29	0.016	0.000–0.036
14	33	0.011	0.000–0.025
15	31	0.012	0.000–0.032
16	29	0.009	0.000–0.022
17	27	0.012	0.000–0.026
19	5	0.010	0.000–0.020
20	26	0.011	0.000–0.028
21	35	0.008	0.000–0.020
22	25	0.012	0.000–0.024
23	28	0.006	0.000–0.015
24	28	0.006	0.000–0.015
26	6	0.007	0.000–0.020
27	29	0.005	0.000–0.017
28	27	0.004	0.000–0.012
29	31	0.004	0.000–0.004

**Figure 2:**
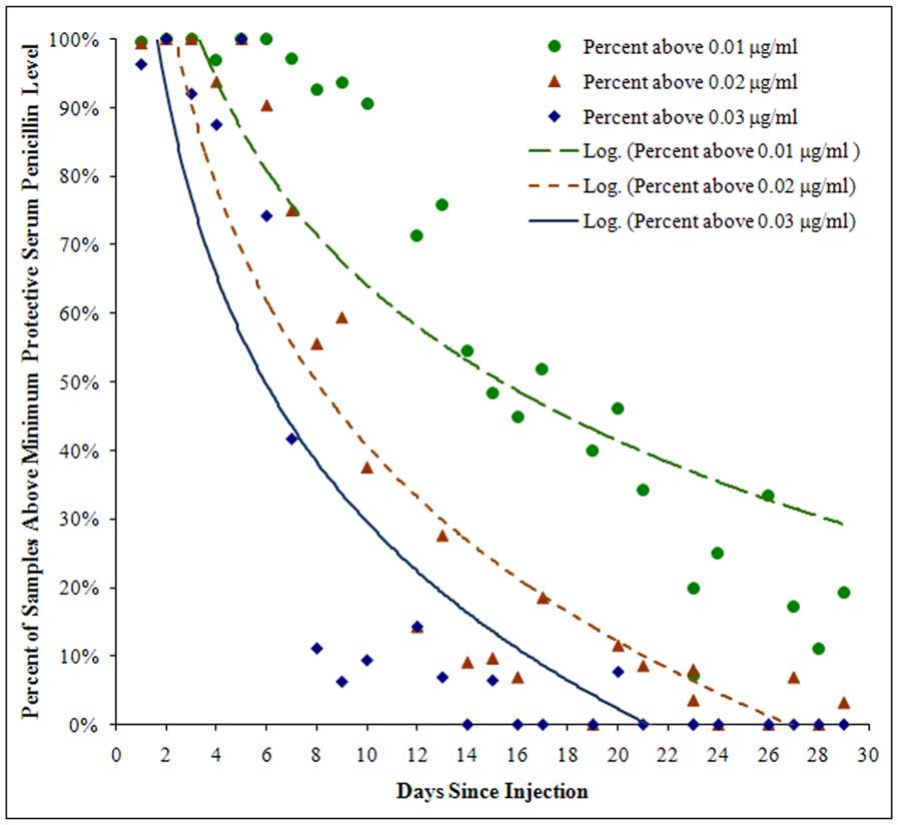
Percentage of subjects whose penicillin G serum level was greater than three proposed minimum protective versus day since injection. Logarithmic trend-line estimates are shown for each protection level. As days progress, the percentage of values above minimum protective per day continually decreases with some day-to-day variation. (Note:day 21 outlier removed from calculation of percentages; see text.)

Figure 2 shows that 50% of the subjects were below 0.03 µg/ml at 6 days, below 0.02 µg/ml at 9 days, and below 0.01 µg/ml at 16 days.

## Discussion

The “protective” penicillin G serum level reflects minimum inhibitory concentration (MIC). Minimum levels of protection have been proposed between 0.01 and 0.03 µg/ml (Broderick, Faix and Hansen, unpublished data). Regardless of the minimum protective concentration adopted (0.01, 0.02, 0.03 µg/ml), serum penicillin G levels in this population of active individuals fall to subprotective levels in less than 2.5 weeks from initial injection. Given the findings of Kaplan and Johnson [12] regarding GAS pharyngitis treatment failures in a pediatric population, and the results of the Bass et al. study [8] showing accelerated declines in serum levels than expected in adults, the results from the present study have important clinical implications.

These results are consistent with those of Bass et al. [8], the only other study on a similar healthy and active young-adult population, and contradict previous studies on persistence of serum penicillin. There are several possible explanations. There could be a difference in the bioavailability of penicillin in the currently manufactured product due to changes in the manufacturing process during the past 50 years. A 1992 study indicated that the duration of adequate serum penicillin G levels varies significantly by manufacturer [13]. To our knowledge, there are no recent studies of the bioavailability of penicillin following transfer of the manufacturing process that occurred in 2008. At that time, the manufacturing process for BPG in the United States was sold by Wyeth Laboratories to Monarch Pharmaceuticals. Despite an intensive effort, we have been unable to find any published detailed description to allow a comparison of the manufactured product when the initial studies were carried out in the early 1950s and the currently available BPG from the successor manufacturer in the United States.

Another important consideration is the activity level of the subjects. Bass et al. [8] reported low serum penicillin levels in military (US Army) trainees, and suggested the possibility that this was due to the active daily routine of these individuals. That study was carried out using the BPG preparation when it was manufactured by the former pharmaceutical company, but those data are generally similar to our findings in the present study. A review of the literature suggests that there may be a difference in penicillin G persistence in studies on people with illness (primarily rheumatic fever/heart disease) and studies on healthy subjects [8], [14], [15], [16].

The clinical implications of our findings are important for the active young adult population, and suggest that a similar study be conducted in children. At issue is the effectiveness of protection given by BPG for rheumatic fever prevention in the weight group we studied if adequate levels are present for only half the duration currently anticipated. In order to maintain adequate levels either the dose or the frequency of dosing should be increased, or both. For example, in a study of patients ranging in age from 16 to 49 years, Currie et al. [7] found that increasing the dose above 1.2 million units of BPG resulted in higher serum penicillin G levels for longer periods of time. If a pharmacokinetic profile similar to the one we have shown in this study is found in children, then we would have the same concerns for adequate protection and appropriate dosing.

These findings also have important implications for the use of BPG in the treatment of and prophylaxis for a variety of other conditions. At the military training facility where this study was conducted, GAS-related respiratory disease is generally well controlled by a BPG injection every 4 weeks. In case of an outbreak, administration of BPG every 2 weeks might be appropriate. Frequency of dosing was increased during a 2002 outbreak of GAS pneumonia along with other interventions such as isolation of ill trainees, cohorting incoming groups, and providing other antibiotics for prophylaxis in penicillin-allergic trainees, which ended the outbreak [17]. As an additional example of the clinical implications of our study, the recommended doses for syphilis treatment are higher than for group A streptococcus-related illnesses (2.4 million units every 7 days). In light of our findings current recommendations for this infection should be re-evaluated not only in adults, but also when treating congenital syphilis in infants.

For treatment of GAS upper respiratory tract infection in children, recent studies have demonstrated failure to eradicate the organism from 35 to 40% of individuals with documented positive throat cultures [12]. Whether this is the result of suboptimal serum penicillin levels or other possibilities such as the streptococcal upper respiratory-tract carrier state remains unknown. Furthermore, it has been re-emphasized that since penicillin does not penetrate well into epithelial cells, lowering the serum-epithelium gradient could well have an adverse effect [18]. These issues are of practical clinical and public health importance and require further study, as well as further consideration in currently available clinical guidelines.

In this population of young healthy adults, penicillin G serum levels degraded more quickly than would have been expected from review of the pharmacokinetics literature. The shorter duration of protective serum levels has implications for the treatment and prevention of diseases related to GAS and other indicated pathogens.
